# Noninvasive evaluation of the skin barrier in reconstructed human epidermis using speckle analysis: Correlation with Raman microspectroscopy

**DOI:** 10.1111/srt.13708

**Published:** 2024-04-17

**Authors:** Léa Habib, Léa Abi Nassif, Marie Abboud, Rime Michael‐Jubeli, Ali Tfayli, Roger Lteif

**Affiliations:** ^1^ Laboratoire d’étude cinétique en milieu hétérogène (LECH) Faculté des Sciences Université Saint Joseph Beirut Lebanon; ^2^ Unité Universitaire Interdisciplinaire Lip(Sys)^2^: Lipides, Systèmes analytiques et biologiques, Faculté de pharmacie Université Paris‐Saclay Orsay France; ^3^ Physics Department, UR TVA, Faculté des sciences Université Saint Joseph Beirut Lebanon

**Keywords:** barrier function, degree of polarization, epidermal models, grain size, lipids, skin research

## Abstract

**Background:**

Reconstructed epidermis models, obtained from 3D keratinocytes culture, have gained significant prominence as prototypes for safety and efficacy testing in skin research. To effectively evaluate these models, it is essential to perform molecular and functional characterization. The skin's barrier function is one of the essential aspects of the epidermis that needs to be assessed. A noninvasive method is thus required for the evaluation of the skin barrier in these models. With this perspective, the aim of this feasibility study is to apply the speckle technique for the assessment of the skin barrier in the Reconstructed Human Epidermis (RHE).

**Materials and methods:**

Speckle analysis as well as Raman microspectroscopy were performed on RHE samples at two maturation days, D17 and D20.

**Results:**

Between D17 and D20, our study showed an increase in various Raman parameters, including stratum corneum percentage, lateral lipid packing, lipid‐to‐protein ratio, and protein secondary structure. Furthermore, the degree of light polarization and the speckle grain size also increased over this period.

**Conclusion:**

The speckle technique proved to be effective for evaluating the skin barrier in Reconstructed Human Epidermis (RHE) models. Comparison with Raman validates this approach and provides comprehensive molecular and functional characterization of reconstructive skin models.

## 1 INTRODUCTION

The human skin, a multifunctional and complex organ, plays a crucial role in protecting the body from external factors, forming a physical barrier. The epidermis, the outermost layer of the skin, acts as a protective barrier against a variety of external aggressions, environmental stress, pathogens, and solar radiation.^1^ Its primary components consist of keratinocytes arranged in a stratified squamous structure. During the process of keratinocyte differentiation, significant changes occur in the lipid composition in order to form the epidermal layers. The outer layer of the epidermis, known as the stratum corneum (SC), is mainly composed of corneocytes. These corneocytes contain a cornified envelope made up of a lipid cement matrix and a cross‐linked sheath comprising a variety of proteins. This structure, known as a “brick and mortar”, plays a critical role in increasing physical strength and maintaining the effectiveness of the skin barrier function.^2^, ^3^

The global movement toward replacing animal testing has prompted the evolution of in vitro skin prototypes as alternatives. The reconstructed human epidermis (RHE), which reproduces the histological structure of its natural equivalent, was initially presented in 1990 by Rosdy and Clauss.^4^ It is created using human primary keratinocytes grown on an inert matrix at the air‐liquid interface. This model consists of organized basal, spinous, and granular layers, along with a multilayered stratum corneum (SC), making it a highly relevant and reliable tool for research and testing purposes.^5^

Thus, the RHE represents a substantial improvement in skin research with a wide range of applications. Researchers can develop more effective treatments for skin diseases and disorders by understanding the mechanisms underlying skin barrier function. In order to characterize the RHE models and evaluate the barrier function, a variety of invasive and noninvasive techniques were used. Protein levels and localization specific to different stages of keratinocyte differentiation were analyzed by immunofluorescence.^6^ The assessment of lipid profiles was conducted by different separation techniques, specifically thin‐layer liquid chromatography and gas chromatography.^7^, ^8^ With the advent of high‐performance liquid chromatography coupled with mass spectrometry (HPLC/MS), a more comprehensive evaluation of the lipid composition becomes possible.^9^, ^10^ Furthermore, the organization of these lipids was evaluated by x‐ray diffraction^11^ and Raman microspectroscopy.^9^, ^10^, ^12^

Raman microspectroscopy is an emerging noninvasive analytical technique in skin research. It is employed in a variety of applications such as in vitro evaluation of skin physiology,^13^, ^14^ analysis of skin penetration studies,^10^, ^15^, ^16^, ^17^ skin diagnosis,^18^, ^19^ as well as in vivo studies.^20^, ^21^, ^22^

Within these contexts, Raman microspectroscopy allows the assessment of skin barrier constituents at a molecular level. It provides valuable data on stratum corneum lipids, natural moisturizing factor components, and protein structural organization.^23^ More recently, Goto et al. used stimulated Raman spectroscopy and imaging for the evaluation of lipids and proteins on skin biopsies.^24^ These methods facilitate the characterization of epidermal barrier dysfunction. In addition, by conducting in‐depth measurements, they enable the analysis of the epidermis penetration of several compounds.^10^, ^25^

Among promising noninvasive technique in skin research, speckle imaging has shown remarkable effectiveness in the in vivo detection of skin cancer.^26^, ^27^, ^28^ Speckle consists in illuminating a medium with a coherent light source, such as a laser, creating speckle patterns of bright and dark spots. These patterns are unique to each scattering medium and can therefore provide information about its characteristics and its dynamics. Research findings indicate that variations in layer thickness of the medium have a direct impact on the changes observed in the degree of light polarization. The backscattered light can be captured by adjusting the orientation of a polarizer to be parallel or perpendicular to the incident light. Thus, the degree of polarization is a valuable parameter for characterizing burns and melanomas.^26^, ^29^

In addition to the up mentioned two parameters, speckle grain size (dx), which refers to the horizontal average size of the bright spots in speckle patterns, is a measure of the spatial scale over which the intensity of the speckle changes providing information about the scattering properties of the illuminated medium. For instance, dx was used in investigating fruit ripening processes, allowing a non‐invasive monitoring of fruit quality attributes such as texture, firmness, and maturity. The alterations in the surface roughness and scattering characteristics can be captured by measuring the speckle grain size.^30^, ^31^

In our study, we intend to evaluate the feasibility of speckle imaging for the evaluation of the skin barrier in RHE models at different maturation days. Simultaneously, information on lipids and proteins global organization as well as the homogeneity of the spatial distribution of the different structures was obtained from Raman microspectrometry. The experimental modifications of speckle parameters were correlated with different Raman ones as well as the information of penetration of caffeine obtained from a study that was previously conducted in the laboratory in the same conditions.^10^

## 2 MATERIALS AND METHODS

### 2.1 Experimental approach for Raman microspectrometry analysis

LabRAM HR Evolution system (Horiba Scientific, Palaiseau, France) was used for the Raman spectral imaging. The measurements were done by a long focal distance microscope objective MPLFLN 100 × (0.90 NA, Olympus) and a 10‐mW power 632.8 nm He–Ne laser. The confocal pinhole was adjusted to 200 µm. The captured light was directed through an edge filter and was then dispersed using a grating of 300 grooves per mm with a spectral resolution of 4 cm^−1^. This dispersion covered a wavenumber ranging from 400 to 3800 cm^−1^.

#### 2.1.1 Raman imaging

For in‐depth Raman imaging, Reconstructed Human Epidermis (RHE) samples were positioned on CaF_2_ slides. We acquired optical cross‐sections (XZ confocal maps) by scanning point by point perpendicular to the surface of the RHE. Stratum corneum percentage was obtained using classical least square fitting.

The experimental protocol and the percentage calculation followed the method described in the study by Bakar et al.^10^

#### 2.1.2 Lipid lateral packing

Additionally, the lipid conformational order was assessed by studying the ratio of *v*_asym_ CH_2_ (2882 cm^−1^) to *v*_sym_ CH_2_ (2852 cm^−1^). This ratio provides valuable insights into the lateral packing and conformational state of lipids. A compact state in lipid packing is relevant to high values of the ratio, indicating a tightly packed arrangement. Conversely, a decrease in the ratio signifies a loosening of the lipid packing, suggesting a less compact state.

#### 2.1.3 Protein structural organization

The analysis of various ratios was conducted to study the protein's structural organization. These ratios include α‐helix (1628–1660 cm^−1^) to Amide I (1608–1700 cm^−1^), β‐sheet (1662–1675 cm^−1^) to Amide I, and (α+β) to Amide I.

### 2.2 Measurement of caffeine percutaneous slope of penetration

**FIGURE 1:**
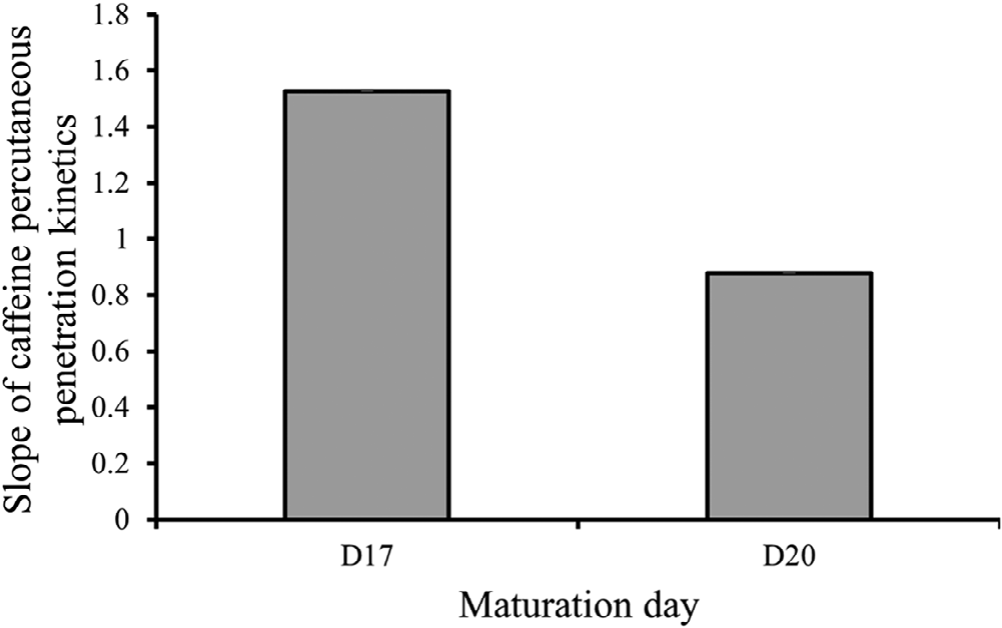
The correlation between the kinetic slopes of caffeine percutaneous penetration and maturation days of the RHE. RHE, reconstructed human epidermis.

For the correlation between the kinetic slopes of caffeine percutaneous penetration and the epidermis thickness during the maturation days of the RHE, the results from a previous study conducted by Bakar et al. were used. As shown in Figure 1, the observed decrease in caffeine percutaneous penetration with the maturation of the RHE is related to the enhancement of the barrier function.

### 2.3 Theoretical and experimental approaches for speckle analysis

#### 2.3.1 Theoretical considerations

Laser speckle imaging has proven to be highly valuable in a wide range of fields, including imaging,^32^ metrology,^33^ and material characterization.^34^ Speckle phenomenon arises when spatially coherent light, such as laser light, interacts with a surface or propagates through a medium.^30^ This interaction leads to the formation of speckle patterns which are characterized by a random arrangement of bright and dark spots. Speckle images serve as a source of information, offering insights into the properties and structure of the illuminated medium. Hence, by analyzing the characteristics of speckle patterns, one can gain a deeper understanding of the behavior and features of the studied diffusing medium.

The speckle image can provide valuable information about polarization characteristics. The response of every illuminated sample to incident light polarization is influenced by its unique dispersion of particle sizes. This distribution affects both the medium's diffusion regime and the polarization of the diffused photons.^29^ This particle size distribution can be inferred by quantifying the polarization characteristics of the backscattered light, specifically through the degree of light polarization. This parameter is determined from measurements of the average speckle intensity using the Equation (1):

(1) $$DOP=\frac{I_{//}-I_{⊥}}{I_{//}+I_{⊥}}$$

where *I*_//_ represents the average intensity measured at the camera surface when the incident light's polarization is completely transmitted and I_⊥_ denotes the average intensity when the transmitted light is a result of a perpendicular polarization (in the case of incident linear polarization), or helicity‐flipped circular polarization (in the case of incident circular polarization). By measuring the Degree of Polarization (DOP) for linear polarization (DOP_L_) and circular polarization (DOP_C_), it becomes possible to evaluate the dominant characteristics of particles or diffusers present in the medium and identify relative changes in their size.^35^

The estimation of the speckle grain size is done by using the normalized auto‐covariance function c_I_(x,y) of the speckle intensity pattern I(x,y), obtained within the camera's observation plane (x,y). It has a zero base, and the width of the function serves as a meaningful measurement for estimating the average width of a speckle grain.^36^ Applying the Wiener‐Khintchine theorem, the calculation of the normalized auto‐covariance function c_I_(x,y) is derived from the intensity distribution of the observed speckle. The expression for the normalized c_I_(x,y) is given by Equation (2):

(2) $$c_{I}\left(x,y\right)=\frac{FT^{-1}\left[|FT\left[I\left(x,y\right)\right]{|}^{2}\right]-{\left(I\left(x,y\right)\right)}^{2}}{\left(I^{2}\left(x,y\right)\right)-{\left(I\left(x,y\right)\right)}^{2}}$$

where *FT* is the Fourier Transform and ⟨⟩ represents a spatial average. The dx dimension, identified as the full width at half maximum (FWHM) of the horizontal profile of *c_I_* (*x,y*), serves as the horizontal size of the speckle grain.

#### 2.3.2 Speckle experimental setup

**FIGURE 2:**
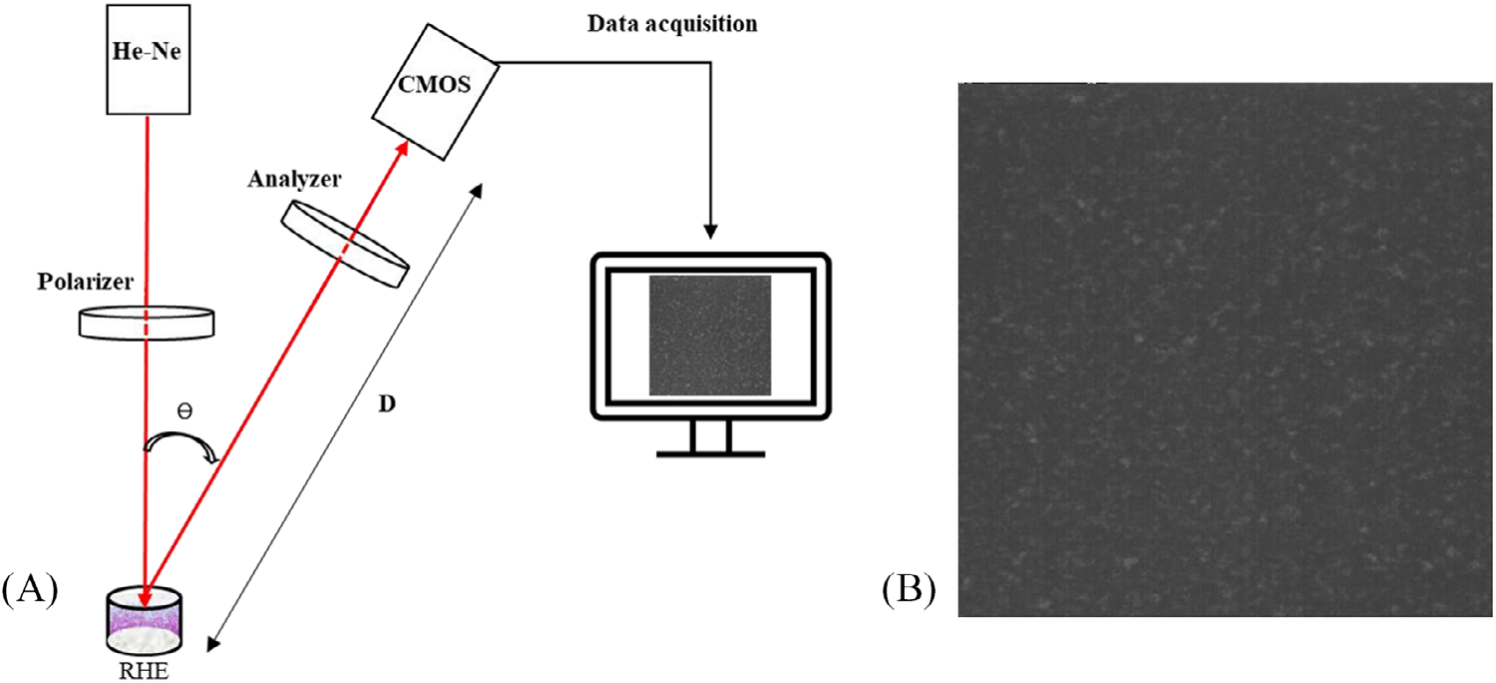
(A) Speckle experimental set‐up and (B) Speckle image (400 pixel × 400 pixel) recorded of the RHE at D20 of maturation. ϴ is the angle between backscattered and incident light; D is the distance between the camera and the RHE sample. RHE, reconstructed human epidermis.

Figure 2 illustrates the speckle arrangement used in our measurements. A 7 mW He‐Ne laser operating at a wavelength of 632.8 nm was linearly polarized using a polarizing cube and directed towards the RHE insert. Backscattered light at an angle ϴ = 20° relative to the incident light direction was captured by a high‐speed recording complementary metal oxide semiconductor (CMOS) camera MotionBlitz EoSens Mini 1 (pixel size of 14 × 14 µm). The exposure time and framerate of the camera were set at 10 ms and 20 frames per second respectively. The camera was positioned at a distance D = 20 cm from the RHE sample in order to ensure a relatively large speckle grain size compared to the pixel size. To control the polarization of the backscattered light detected by the camera, an analyzer was placed in front of the camera. When the analyzer's axis aligned with that of the polarizing cube, the camera detected backscattered light with polarization parallel to that of the incident light. Conversely, when the analyzer's axis was perpendicular to that of the polarizing cube, the camera detected backscattered light with polarization perpendicular to that of the incident light.

Speckle measurements were conducted on RHE samples at two different maturation days (D17 and D20). For each RHE sample, a series of speckle images with dimensions of 400 pixels by 400 pixels were recorded. These speckle images were then spatially analyzed. The analysis was carried out for the linear parallel and linear perpendicular polarizations of the backscattered light.

### 2.4 Statistical analysis

To assess the statistical significance of the difference between the two groups, we conducted a *t*‐test.

## 3 RESULTS

**FIGURE 3:**
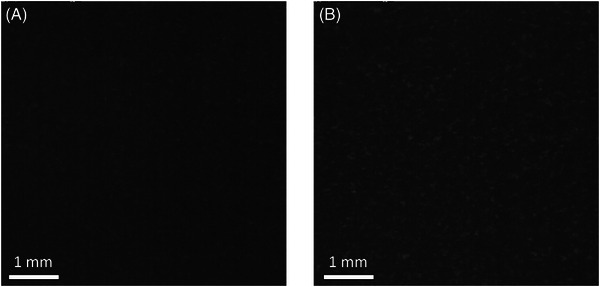
Examples of speckles image recorded of the RHE at (A) D17 and (B) D20 of maturation. RHE, reconstructed human epidermis.

During the maturation process, the epidermis, therefore the skin barrier, undergoes several changes, resulting in an improved skin barrier function. For the reconstructed human epidermis (RHE) undergoing maturation, examples of recorded speckle images at different maturation days (D17 and D20) are shown in Figure 3. Speckle grain size dx and DOP_L_ were computed for each sample.

In parallel, Raman microspectroscopy allowed to monitor the evolution of the SC at the surface of RHE, the lipids to proteins ratio, the protein secondary structures, the lateral packing of lipids and the homogeneity of their distribution.

Finally, speckle parameters and the molecular composition as well as the organization obtained from Raman microspectroscopy were correlated.

### 3.1 Impact of maturation day on lipid structural aspect

**FIGURE 4:**
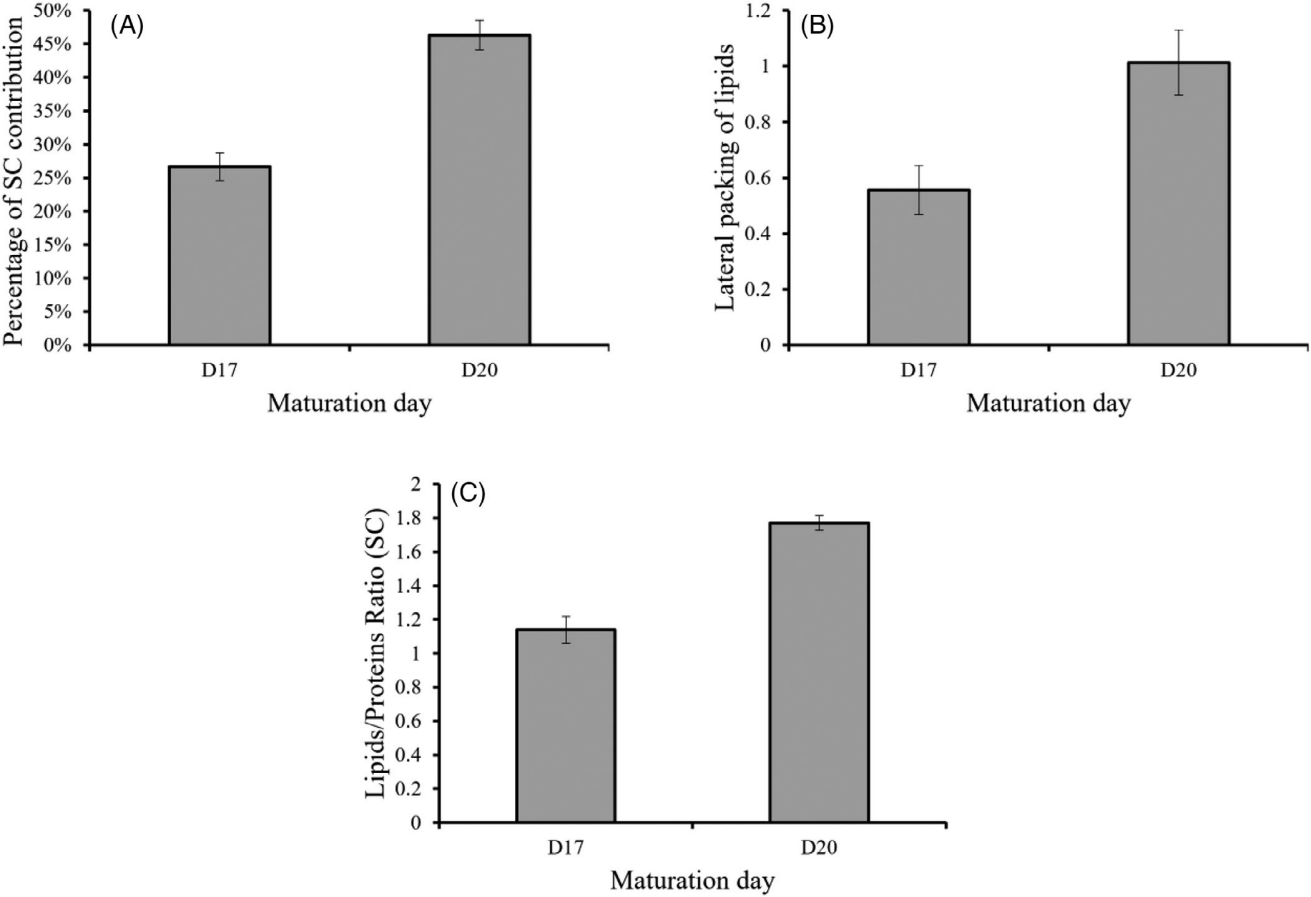
Variation of (A) the percentage of stratum corneum (SC) contribution, (B) the lateral packing of lipids, and (C) the lipids‐to‐proteins ratio within the SC of the RHE during different maturation days. RHE, reconstructed human epidermis; SC, stratum corneum.

In this section, we present results related to the progression of stratum corneum (SC), as well as lateral packing of lipids, and the ratio of lipids to proteins throughout the maturation process of RHE. As shown in Figure 4, there is a noticeable increase in the percentage of the SC within the RHE as the maturation process advances. The SC represents the most organized structure of the epidermis. It is composed of layers of corneocytes embedded in a lipid matrix. This lipid matrix acts as a cement, holding the corneocytes together and maintaining the integrity and organization of the SC. Therefore, an increase in its percentage leads to a more structured and organized surface of the RHE, as indicated in Figure 4. We observed that the lateral packing of lipids increases with the differentiation process. This could also be associated with higher lipid content within the SC, thus explaining the observed increase in the lipid‐to‐protein ratio.

### 3.2 Impact of maturation day on protein organization in the SC

On the other hand, we analyzed the ratio between the Amide I band (1608–1700 cm^−1^), which represents the total protein content, and the secondary structures of the proteins, specifically the α‐helix (1628–1675 cm^−1^) and the β‐sheet (1662–1675 cm^−1^), as well as the ratio to their combined sum. The α‐helix structure is known for its high stability with limited exposure of sidechains, while β‐sheet is smoother and possesses a greater number of exposed sidechains between the sheets.^23^

**FIGURE 5:**
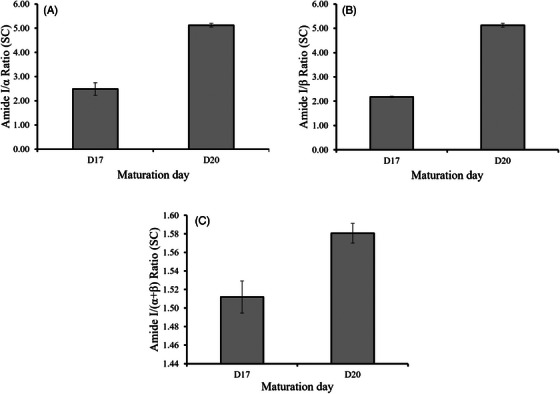
Variation of (A) Amide I/α, (B) Amide I/β, and (C) Amide I/(α+β) ratios within the SC of the RHE during different maturation days. RHE, reconstructed human epidermis; SC, stratum corneum.

As the stratum corneum (SC) matures, the secondary structure of the proteins within it becomes more ordered and structured (Figure 5). This is evident in the increase of the studied ratios between day 17 and day 20. This improved organization and structure of the proteins leads to a stronger and more cohesive barrier, making the skin more resistant to damage and improving its overall health.

### 3.3 Impact of maturation day on the degree of polarization

**FIGURE 6:**
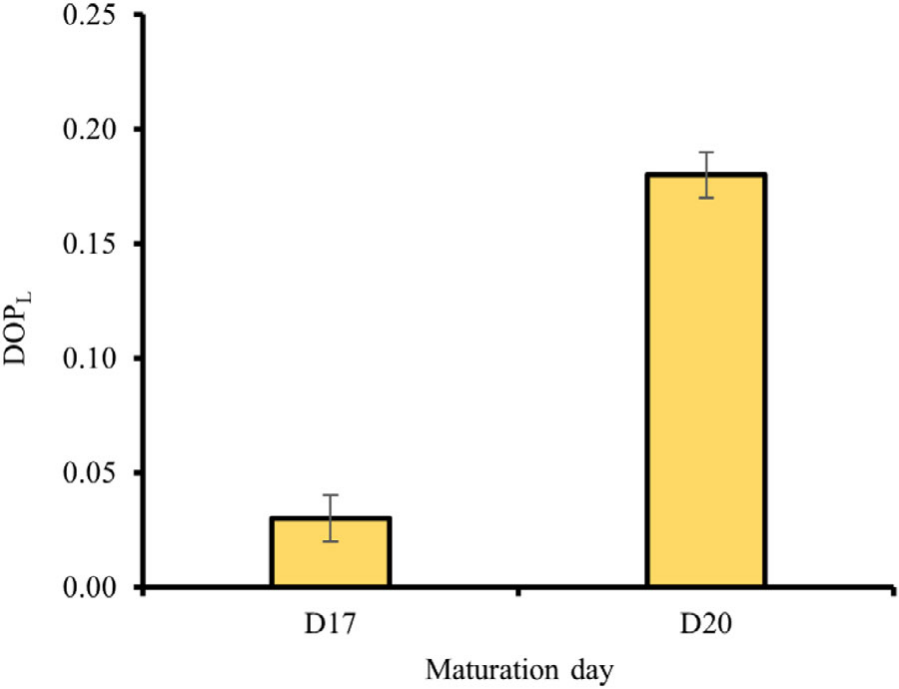
Variation of DOPL within the RHE during maturation days. Error bars are the standard deviations. DOPL, degree of liner polarization; RHE, reconstructed human epidermis.

To thoroughly evaluate the scattering properties of RHE throughout the maturation and determine the dominant type of backscattered photons, the degree of light polarization is measured. Figure 6 shows that the linear polarization degree (DOP_L_) increases from D17 to D20.

The statistical analysis revealed a significant difference among the DOP_L_ values (*p* = 9.05 × 10^−8^, *p* < 0.05). We noticed an approximate increase of 432% when comparing DOP_L_ at days 17 and 20 of maturation.

The increased degree of light polarization suggests a more ordered or aligned structure, possibly due to enhanced organization of tissue elements.

### 3.4 Impact of maturation day on speckle grain size

The biochemical structure of the epidermis, as well as the microstructure of the stratum corneum (SC) in RHE, changes significantly as it matures. Analyzing the speckle grain size (dx) could potentially provide valuable information about these characteristics. Therefore, we also evaluated the variation of dx across D17 and D20. A significant difference was observed among the two dx values (*p* = 3.30 × 10^−6^, *p* < 0.05).

**FIGURE 7:**
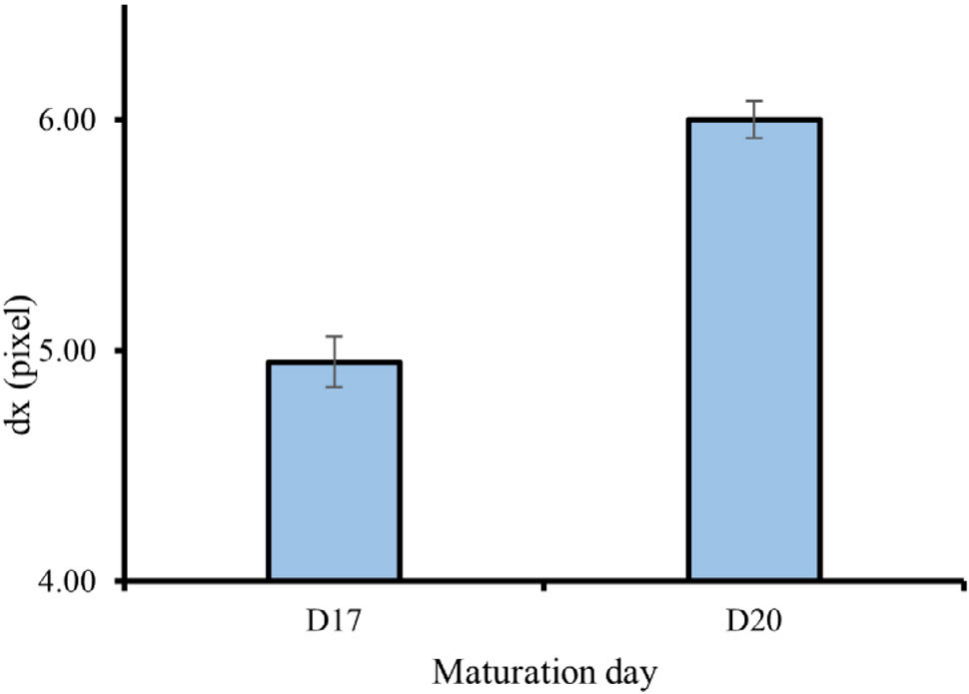
Variation of horizontal speckle grain size within the RHE during maturation days. RHE, reconstructed human epidermis.

Results, illustrated in Figure 7, show that there was an increase of around almost 1 pixel when comparing dx on D17 and D20.

Similar to changes in degree of light polarization, the increase in dx suggests modifications in the scattering properties of the RHE. This might be indicative of the emergence of well‐defined structures with a more uniform orientation as well as an increase of RHE density as cells become more tightly packed and organized.

## 4 DISCUSSION

Given the objective of our study, which is mainly the feasibility of skin barrier assessment using the speckle imaging technique, we chose to use a maturation period of just 2 days for RHE samples. This would allow us to obtain valuable preliminary information and results, while taking into consideration the practical limitations imposed by the availability of these samples.

**FIGURE 8:**
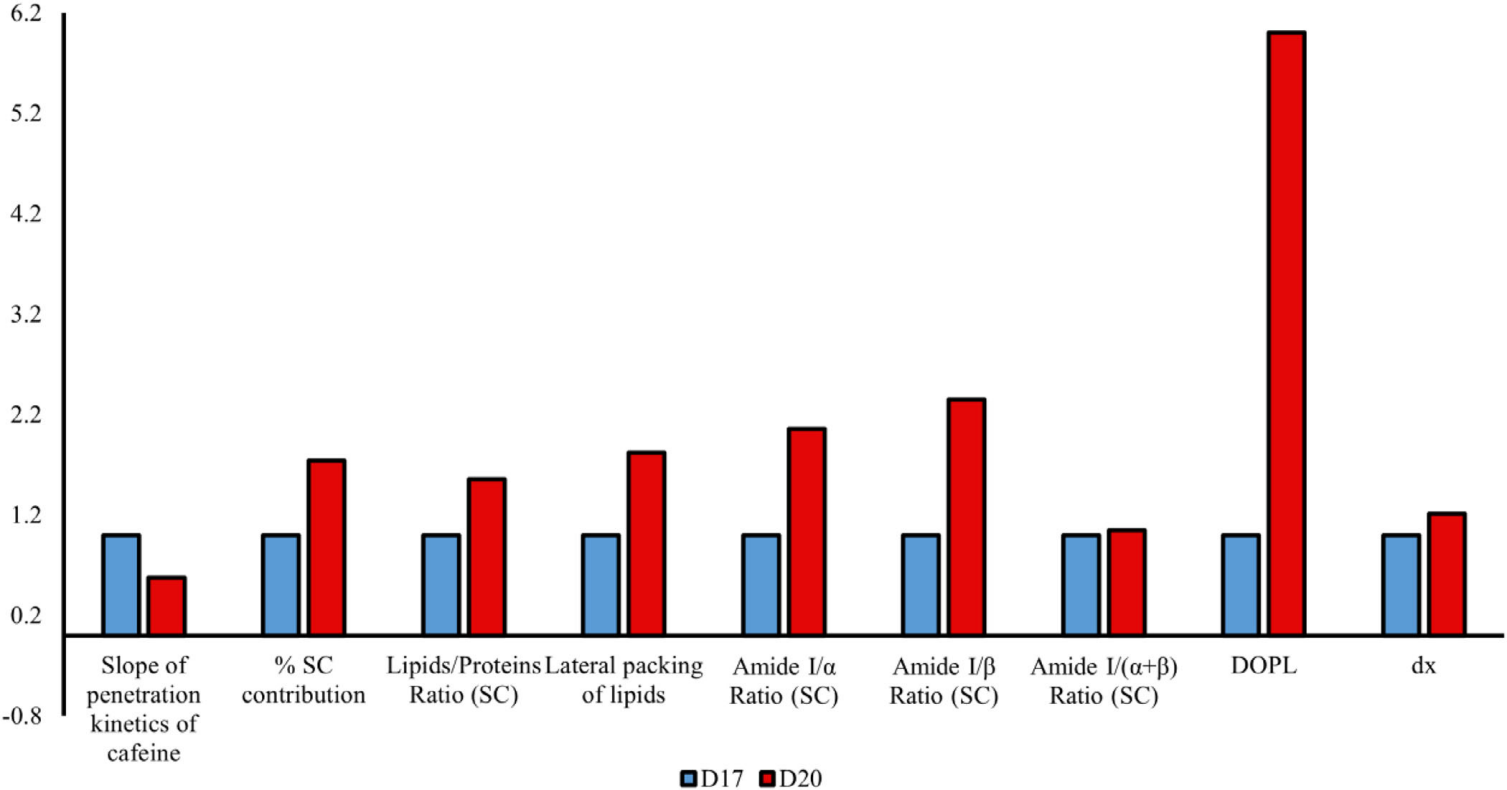
Variation of Raman and Speckle parameters between days 17 and 20 of RHE maturation. RHE, reconstructed human epidermis.

As illustrated in Figure 8, we observe that Raman and Speckle parameters evolve consistently between D17 and D20. Thus, the variations in Raman parameters during RHE maturation can explain the observed changes in Speckle parameters.

The SC, the outermost layer of the epidermis, undergoes significant changes during maturation.

The maturation of the RHE between D17 and D20 leads to variations in the concentration and organization of different components, mainly lipids and proteins in the stratum corneum.

The SC is the highly organized structure of the epidermis, consisting of corneocytes surrounded by a lipid matrix ensuring the compactness and structure of the SC. A higher percentage of the SC leads to a more structured surface, as well as higher lipid values, which aligns with the higher DOP_L_ values. Furthermore, higher lateral packing leads to increased ordered and aligned lipid structures larger in size compared to disordered domains, therefore less randomization of polarization directions. The uniform orientation of packed lipid structures scatters light coherently compared to disordered structures. All of this contribute to better preservation of polarized light and thus an increase in DOP_L_ during RHE maturation.

Additionally, the observed increase in the percentage of the SC suggests a higher abundance of corneocytes, which are known to contain keratin filaments and act as large scatterers. This could as well be correlated with the increased values of DOP_L_ and dx.

From another perspective, an anti‐correlation of the DOP_L_ with the slope of caffeine penetration was found. The slope of caffeine penetration provides information about the state of the barrier function. High slope values indicated a high rate of penetration, indicating a looser barrier function, which is associated with the looser organization in the SC.

Besides, the increase in protein order parameters (Amide I/α, Amide I/β, and Amide I/(α+β) ratios in the SC) reflects changes in light absorption and scattering by proteins, leading to an increase in the speckle grain size. The α‐helix structure is highly stable with limited exposure of sidechains, making it ideal for forming a strong and cohesive barrier. The β‐sheet structure is smoother and has a greater number of exposed sidechains between the sheets. While this makes it less stable than the α‐helix structure, it also gives it more flexibility. This flexibility may be important for allowing the SC to adapt to changes in the environment and maintain its barrier function.

Consequently, the formation of more ordered, tightly packed, and layered structures enriched in aligned lipids and proteins during maturation enables the propagation of the optical field over larger distances, increasing the speckle grain size.

To conclude, the observed increase in the DOP_L_ and in the dx with maturation days likely reflects the evolving structural and biochemical characteristics of the RHE in terms of cellular differentiation, SC development as well as lipid‐to‐protein ratio. These changes are related to more organized RHE structures. These information may be completed with other techniques such as AFM and SEM which would give valuable information on the proteins intensity and density.^37^

## 5 CONCLUSION

We performed speckle images on RHE during two different maturation days. Our study showed a modification in parameters within the spatial analysis of speckle patterns. The correlation between the speckle results and Raman microspectroscopy enhanced our ability to comprehensively characterize the molecular properties and structural characteristics of these models. Therefore, validating speckle imaging technique as a method for assessing skin barrier function.

This advancement could have significant implications for the development and refinement of skin research methodologies, promoting safer and more efficacious testing of cosmetic and pharmaceutical products.

## AUTHOR CONTRIBUTIONS

Léa Habib: Conceptualization; acquisition, analysis and interpretation of data; validation; writing original draft. Léa Abi Nassif: Supervision; acquisition and analysis of data. Marie Abboud: Supervision; visualization; methodology; analysis and interpretation of data; validation, partial contribution to the writing of the manuscript. Rime Michael‐Jubeli: Supervision; visualization; methodology; analysis and interpretation of data; validation, partial contribution to the writing of the manuscript. Ali Tfayli: Supervision; conceptualization; project administration; methodology; analysis and interpretation of data; validation; writing review, editing and final approval. Roger Lteif: Supervision; conceptualization; project administration; methodology; analysis and interpretation of data; validation; writing review, editing and final approval. All authors have read and accepted the final version of the manuscript for publication.

## Data Availability

The data that support the findings of this study are available from the corresponding author upon reasonable request.
